# Mechanisms of Adaptation from a Multiple to a Single Step Recovery Strategy following Repeated Exposure to Forward Loss of Balance in Older Adults

**DOI:** 10.1371/journal.pone.0033591

**Published:** 2012-03-16

**Authors:** Christopher P. Carty, Neil J. Cronin, Glen A. Lichtwark, Peter M. Mills, Rod S. Barrett

**Affiliations:** 1 Centre for Musculoskeletal Research, Griffith Health Institute and School of Physiotherapy and Exercise Science, Griffith University, Gold Coast, Australia; 2 Department of Biology of Physical Activity, University of Jyväskylä, Jyväskylä, Finland; 3 School of Human Movement Studies, The University of Queensland, Brisbane, Australia; University of Zaragoza, Spain

## Abstract

When released from an initial, static, forward lean angle and instructed to recover with a single step, some older adults are able to meet the task requirements, whereas others either stumble or fall. The purpose of the present study was to use the concept of margin of stability (MoS) to investigate balance recovery responses in the anterior-posterior direction exhibited by older single steppers, multiple steppers and those that are able to adapt from multiple to single steps following exposure to repeated forward loss of balance. One hundred and fifty-one healthy, community dwelling, older adults, aged 65–80 years, participated in the study. Participants performed four trials of the balance recovery task from each of three initial lean angles. Balance recovery responses in the anterior-posterior direction were quantified at three events; cable release (CR), toe-off (TO) and foot contact (FC), for trials performed at the intermediate lean angle. MoS was computed as the anterior-posterior distance between the forward boundary of the Base of Support (BoS) and the vertical projection of the velocity adjusted centre of mass position (XCoM). Approximately one-third of participants adapted from a multiple to a single step recovery strategy following repeated exposure to the task. MoS at FC for the single and multiple step trials in the adaptation group were intermediate between the exclusively single step group and the exclusively multiple step group, with the single step trials having a significant, 3.7 times higher MoS at FC than the multiple step trials. Consistent with differences between single and multiple steppers, adaptation from multiple to single steps was attributed to an increased BoS at FC, a reduced XCoM at FC and an increased rate of BoS displacement from TO to FC. Adaptations occurred within a single test session and suggest older adults that are close to the threshold of successful recovery can rapidly improve dynamic stability following repeated exposure to a forward loss of balance.

## Introduction

Falls in older adults are a significant public health concern with approximately one in three community dwelling older adults falling each year [1]. Falls can result in serious injuries, leading to long term disability and in some cases death [2]. The reasons for the high incidence of falls in older adults are complex and multifaceted [3], but may be better understood by examining factors that influence balance recovery capacity in response to a postural perturbation. One experimental approach for studying balance recovery is the tether-release method [4], which involves tilting a participant into a static forward lean position via the use of a horizontal tether, that is subsequently released after a random time delay. Using this experimental approach it has been shown that older adults have a lower maximum lean angle from which they can recover with a single step [5] and are more likely to require multiple steps to recover from a given lean angle than young adults [6], [7]. The tendency to use multiple steps to recover from loss of balance is also predictive of a future fall [8], and so it follows that it is important to identify the mechanisms that influence the ability to recover from loss of balance in older adults.

Older adults have been shown to exhibit adaptive and/or reactive adaptations in their balance recovery behaviour in response to repeated exposure to the balance recovery task [9], [10], [11]. Barrett et al. [12] used the tether-release method to induce forward loss of balance in older adults, and quantified adaptive stepping responses using the concept of margin of stability [13]. Within the paradigm of margin of stability, an individual can theoretically improve their dynamic stability during recovery from a forward loss of balance by taking longer, faster steps, adopting a posture in which the whole body centre of mass shifted posteriorly and/or reducing anterior centre of mass velocity. Indeed, the margin of stability when the stepping foot touches down is strongly predictive of the recovery strategy employed (i.e., single versus multiple steps) [7]. Barrett et al. [12] found that, on repeated exposure to the task within a single balance recovery test session, older adults exhibited improvements in anterior-posterior, but not medial-lateral margin of stability. The primary mechanism underlying the observed improvement in margin of stability at foot contact with repeated task exposure in the study by Barrett et al. [12] was a reduction in the anterior-posterior position and velocity of the whole body centre of mass. However participants in this study consisted of three main sub-groups: individuals who were able to recover balance with a single step as instructed (i.e., single steppers); individuals who required multiple steps or support from an overhead harness system to recover balance (i.e., multiple steppers); and individuals who were unable to recover with a single step on initial exposure to the task, but learned to recover with a single step in subsequent trials (i.e., mixed steppers). Since Barrett et al. [12] did not attempt to differentiate between subgroups within their study on the basis of the recovery strategy employed, it remains possible that subgroups within their participant sample exhibited differences in the nature and extent of their adaptive responses. The mechanisms by which older adults that can rapidly learn to recover balance are currently unknown, and if identified, could provide insight into ways in which balance recovery of older adults could be improved more generally.

The purpose of the present study was to use the concept of margin of stability to investigate balance recovery responses in the anterior-posterior direction exhibited by older single, multiple and mixed steppers following exposure to repeated forward loss of balance. It was hypothesised that the margin of stability would be improved in trials where mixed steppers were able to recover with single compared to multiple steps and that the mechanism of improvement in margin of stability exhibited by the mixed group would be consistent with the mechanisms that differentiate the exclusively single from the exclusively multiple step groups.

## Methods

### Participants

One hundred and fifty-one healthy, community dwelling older adults (79 male, 72 female), aged 65–80 years, were recruited at random from the local electoral roll. The mean (±1 SD) age, height and mass of participants were 71.6±4.6 years, 1.67±0.09 m and 75.8±12.8 kg, respectively. Individuals that reported neurological, metabolic, cardio-pulmonary, musculoskeletal or uncorrected visual impairment were excluded. Ethics approval was obtained from the Griffith University Human Research Ethics Committee. Written informed consent was obtained from participants prior to testing and methodology was conducted in accordance with the Declaration of Helsinki.

### Balance recovery protocol

The balance recovery protocol was conducted as described previously [12]. Participants stood barefoot with their feet shoulder-width apart in a neutral posture and were tilted forward, with their feet flat on the ground, until 15, 20 or 25% of body weight (BW) was recorded on a load cell (S1W1kN, XTRAN, Australia) placed in series with an inextensible cable. One end of the cable was attached to a safety harness worn by the participant at the level of their sacrum and the other end was attached to a rigid metal frame located behind the participant. An electric winch mounted on the frame was used to adjust the length of the cable. Care was taken to ensure the cable was parallel with the ground and that participants kept their head, trunk and extremities aligned prior to cable release. The cable was released at a random time interval (2–10 s) following achievement of the prescribed posture and cable force (±1%BW), through the disengagement of an electromagnet located in-series with the cable. Participants were instructed to relax their muscles while leaning and to regain balance with a single step using the stepping lower limb of their choice. The instruction to attempt to recover using a single step was communicated to the participant prior to every trial. A second cable, instrumented with a load cell (S1W1kN, XTRAN, Australia), attached the safety harness to the ceiling, and was used to prevent participants from contacting the ground in the event of a fall. Overhead cable force and centre of pressure location were displayed in real time on a computer monitor and were visually inspected by the investigator to ensure anticipatory actions (e.g., anterior-posterior and medial-lateral weight shifting) were not evident in the period immediately prior to cable release. Following an initial trial at the 15%BW lean magnitude, participants performed 4 trials at each of 3 lean magnitudes in random order. All analysis was confined to the trials performed at the 20% lean magnitude condition, which corresponded to a forward lean at cable release of 13.7±1.9 degrees, as measured by the rotation of the whole body centre of mass about the ankle joint centre.

### Data collection and analysis procedures

Trajectories of 51 reflective markers attached to the head, trunk, pelvis, and upper and lower limbs were recorded at 200 Hz using a 10-camera, three-dimensional motion capture system (Vicon MX cameras, Vicon Motion Systems, Oxford, UK) and processed using Vicon Nexus software (Version 1.4, Vicon Motion Systems, Oxford, UK). Full details regarding marker placement and the model for computing the whole body centre of mass kinematics are provided in Barrett et al. [12]. Ground reaction force (GRF) data were simultaneously acquired at 1 kHz using two 900×600 mm piezoelectric force platforms (Type 9287A, Kistler Instrument Corporation, USA). A single force platform was located under both feet at cable release, and a second force platform was located anterior to the first platform to record ground reaction forces from the stepping foot following foot contact.

The criteria used to distinguish a multiple from single step recovery strategy for each trial were: (1) a second step of any kind by the stepping leg or anterior progression of the non-stepping foot past the stepping foot following the initial step [14], and (2) application of 20% BW or more to the ceiling restraint cable at any point during the second step [15]. The following groups were subsequently defined on the basis of the recovery strategy adopted by each participant across trials:

1. Single steppers. Participants in this group used a single step recovery strategy for all 4 trials.
2. Mixed steppers. Participants in this group used a multiple step recovery strategy on some trials and a single step strategy on other trials. For analysis purposes, the trials in this group were subdivided as follows:
  - Mixed-single steppers. This subgroup consisted of trials (up to 3) performed by mixed steppers where a single step strategy was adopted.
  - Mixed-multiple steppers. This subgroup consisted of trials (up to 3) performed by mixed steppers where a multiple step strategy was adopted.
3. Multiple steppers. Participants in this group used a multiple step recovery strategy for all 4 trials.

The Margin of Stability (MoS) in the anterior-posterior direction was calculated using MoS = BoS – XCoM. The Base of Support (BoS) is an estimate of the range of positions that the centre of pressure of the net ground reaction force is confined to act within. The BoS in the antero-posterior direction was defined as the horizontal distance from the great toe marker on the rear leg to the corresponding marker on the step leg. The extrapolated centre of mass position (XCoM) was obtained using 
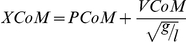
, [13] where PCoM in the antero-posterior direction is the anterior-posterior distance from the great toe marker on the rear foot to the vertical projection of the centre of mass, VCoM is the velocity of the whole body centre of mass, *g* is the acceleration due to gravity and *l* is the effective pendulum length in the sagittal plane. Specific detail of how each MoS-related parameter was defined and computed in the present study is provided in Barrett et al. [12].

MoS and parameters used to compute MoS were assessed at 3 events defined as follows: (1) Cable Release (CR): a 5% reduction in force measured using a force transducer in series with the restraining cable, (2) Toe Off (TO): defined from the vertical motion of the great toe marker on the stepping foot [16] and (3) FC: a GRF in excess of 5 N recorded on the anterior force plate. Average rate of BoS displacement from TO to FC (BoS rate), and the duration, average anterior-posterior GRF reaction force and associated impulse generated by the stepping and non-stepping feet for the period CR to TO and by the non-stepping foot for the period TO to FC, were also computed.

### Statistical analysis

Analysis of Covariance (ANCOVA) was used to assess the effect of group (4 levels: Single steppers, Mixed-single steppers, Mixed-multiple steppers, Multiple steppers) on the dependent measures at the 20% body weight lean angle only, which has previously been shown to divide older adults into approximately equal sized groups with a positive versus negative antero-posterior MoS at FC [7]. Covariates were age, height and body mass. A-priori contrasts were performed to assess differences between adjacent groups and between single and multiple steppers. Significance was accepted for *p*<0.05.

## Results

### Stepping strategies and participant characteristics

No differences in horizontal cable force or forward lean angle at CR between groups were detected across repeated trials at the 20% lean magnitude (*p*>0.05). Of the 151 participants, 43 (29 males, 14 females) were classified as single steppers (Age: 70.1±3.8, Height: 1.68±0.09 m, Mass: 74.5±10.9 kg), 52 (28 males, 24 females) as mixed steppers (Age: 70.9±3.6, Height: 1.68±0.09 m, Mass: 77.5±14.0 kg) and 56 (22 males, 34 females) as multiple steppers (Age: 73.3±5.3, Height: 1.66±0.08 m, Mass: 75.5±13.1 kg). There was a significant main effect of group on age (F = 7.19, *p* = 0.01). Planned contrasts revealed multiple steppers were significantly older than single steppers (*p*<0.01) and mixed steppers (*p* = 0.02). Within the mixed group, 56% of participants adapted from multiple to single step strategy following the initial trial, 25% following the second trial and 19% following the third trial. Sixteen of the fifty-two participants within the mixed group reverted back to a multiple step strategy after successfully performing one or two single step recoveries.

### Margin of Stability (MoS)

**Figure 1:**
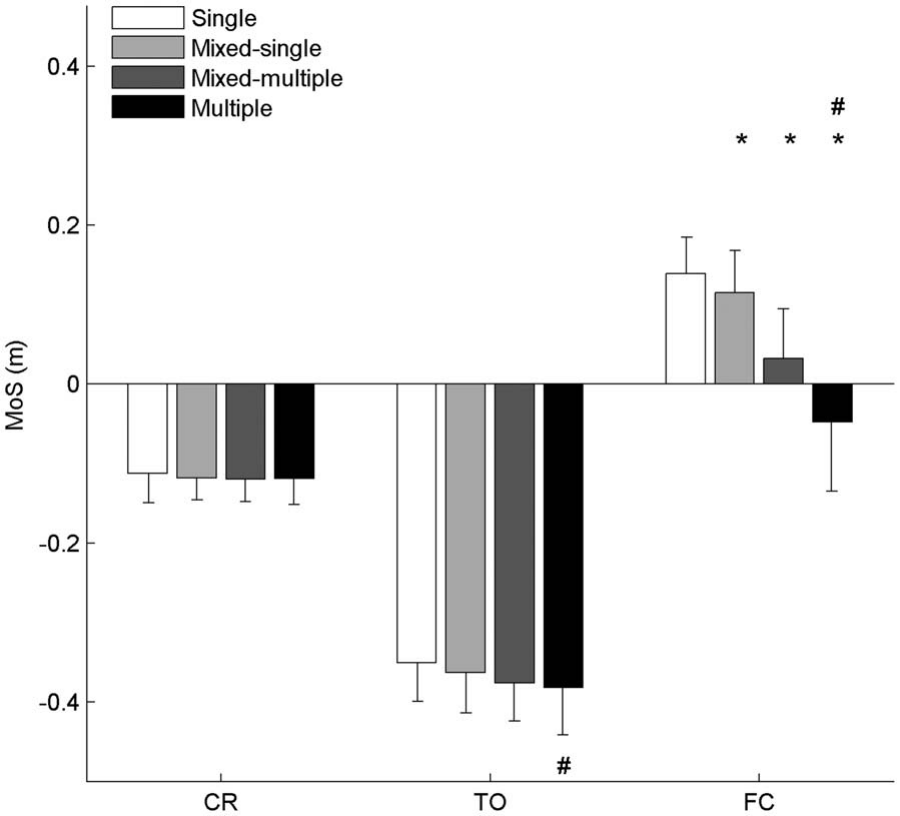
Margin of Stability (MoS) for each group at each event. CR = Cable Release, TO = Toe Off, FC = Foot Contact. The asterisk symbol (*) indicates significant difference with respect to the previous group. The hash symbol (#) indicates significant difference with respect to single steppers.

**Figure 2:**
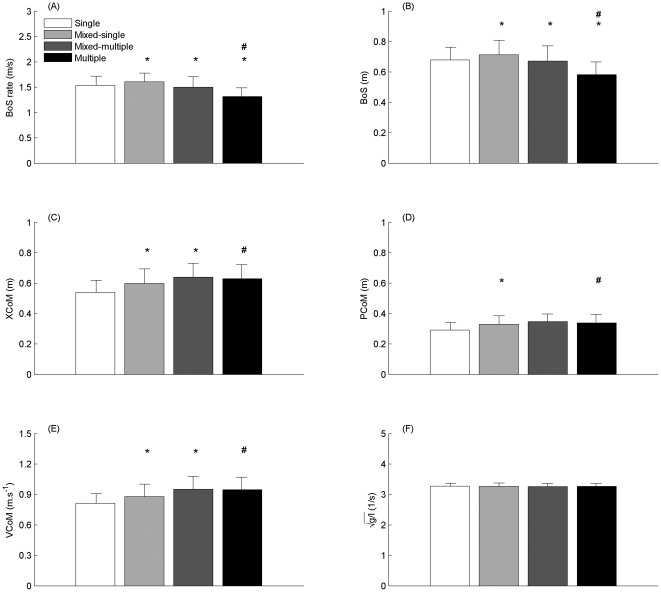
Margin of Stability (MoS) parameters at Foot Contact (FC) for each group. BoS = Base of Support, XCoM = Extrapolated Centre of Mass, PCoM = vertical Projection of the Centre of Mass, VCoM = Velocity of the Centre of Mass, BoS rate = average rate of BoS diaplacement from toe-off to foot-contact, *g* = acceleration due to gravity and *l* = pendulum length in the sagittal plane. The asterisk symbol (*) indicates significant difference with respect to the previous group. The hash symbol (#) indicates significant difference with respect to single steppers.

Group had a significant main effect on MoS at TO (F_4_ = 6.41, *p*<0.01) and FC (F_4_ = 75.18, p<0.01). Planned contrasts revealed significant differences between single and multiple steppers for MoS at TO (*p*<0.01), and between all groups for MoS at FC (*p*<0.05) (Figure 1). At FC, group had a significant main effect on BoS (F_4_ = 16.65, p<0.01), XCoM (F_4_ = 14.57, *p*<0.01), PCoM (F_4_ = 12.00, *p*<0.01), VCoM (F_4_ = 16.98, *p*<0.01). BoS rate was also significantly different between all groups (F_4_ = 26.23, *p*<0.01). Planned contrasts revealed significant differences between groups as depicted in Figure 2. Mixed-single steppers had greater BoS rate, BoS, XCoM, PCoM and VCoM compared to single steppers. Mixed-multiple steppers had lesser BoS rate and BoS, and greater XCoM and VCoM compared to mixed-single steppers. Multiple steppers had lesser BoS rate and BoS compared to mixed-multiple and single steppers. Multiple steppers also had greater XCoM, PCoM and VCoM compared to single steppers.

### Durations between events, GRF and impulse

Group had a significant main effect on the duration, mean anterior-posterior GRF and impulse generated by the stepping and non-stepping feet for the period CR to TO and by the non-stepping foot for the period TO to FC. Planned contrasts revealed significant differences between groups as presented in Table 1. Mixed-single steppers had increased anterior-posterior GRF and impulse from CR to TO and from TO to FC compared to single steppers. Mixed-multiple steppers increased anterior-posterior GRF and impulse from TO to FC compared to mixed-single steppers. Multiple steppers had a lesser duration from CR to TO and increased anterior-posterior GRF and impulse from TO to FC compared to single steppers.

**Table 1:** Temporal and rear foot ground reaction force variables by group (Mean ± SD).

	Single	Mixed-single	Mixed-multiple	Multiple	F, *p* (ANOVA)
CR-TO duration (s)	0.27±0.05	0.26±0.03	0.27±0.03	0.28±0.04	3.63, 0.01
TO-FC duration (s)	0.19±0.04	0.19±0.03	0.19±0.03	0.18±0.02#	3.13, 0.03
Mean A-P ground reaction force CR-TO (N)	134±33	158±39*	156±45	141±41	4.69, <0.01
Mean A-P ground reaction force TO-FC (N)	95±37	125±33*	147±44*	145±47#	19.59, <0.01
Mean A-P Impulse CR-TO (N.s)	34±9	40±10*	40±11	38±11	2.99, 0.03
Mean A-P Impulse TO-FC (N.s)	18±8	24±8*	27±10*	24±9#	10.37, <0.01

CR = Cable Release, TO = Toe Off, FC = Foot Contact. A-P = Anterior-Posterior.

*Significant difference with respect to previous group.

# Significant difference with respect to single steppers.

## Discussion

No group differences in MoS were detected at CR suggesting that the initial stability conditions were effectively controlled through a combination of prior instructions to participants, real-time monitoring of cable force and centre of pressure data, and use of a random time delay prior to CR. However, significant main group effects were detected for MoS at TO, and most notably at FC, where all group comparisons conducted were significant. Approximately one-third of participants were able to alter their recovery response from multiple to single steps following repeated exposure to forward loss of balance and were subsequently classified as using a mixed strategy. In support of our hypothesis, the MoS was improved in trials where mixed steppers were able to recover with single compared to multiple steps. The MoS at FC for mixed-single steppers and mixed-multiple steppers were intermediate between the exclusively single step group and the exclusively multiple step group, with mixed-single steppers having a significant, 3.7 times higher MoS at FC than mixed-multiple steppers. In contrast to multiple steppers, mixed-multiple steppers had a positive MoS at FC, suggesting that these participants were closer to the threshold of recovery with a single step than those in the exclusively multiple step group. The finding that multiple steppers were significantly older than the mixed and single steppers suggests factors associated with ageing such as declines in muscular strength and neuromotor control may contribute to the inability to recover with a single step. The MoS for mixed-single steppers reached only 83% of the MoS observed in the single step group. Therefore, while further task exposure could lead to further improvements in MoS in the mixed and multiple step groups, other factors, such as muscle weakness may limit the ability to recover with a single step. The finding that 16 out of 52 participants (∼31%) in the mixed group reverted to a multiple step strategy following one or two single step trials further reinforces the suggestion that the mixed group were operating close to the threshold for recovery with a single step, where small differences in the recovery response can influence the ability to recovery with a single step. However, we cannot discount the possibility that fatigue was a factor that led to the lapse back to a multiple step strategy in these 16 participants.

Findings from the present study also support our hypothesis that the mechanism of improvement exhibited by the mixed group in single versus multiple step trials was consistent with differences between exclusively single versus multiple step groups. In agreement with the findings of Arampatzis et al. [14], the greater MoS at FC in the exclusively single compared to the exclusively multiple step group were attributed to greater BoS and BoS rate, and lesser XCoM, PCoM and VCoM. The greater MoS at FC for the mixed-single versus mixed-multiple steppers was consistent with differences between exclusively single and multiple step groups and was explained by a corresponding increase in BoS and BoS rate, as well as a decrease in XCoM brought about by a decrease in VCoM. The decreased VCoM in mixed-single steppers was in turn explained by a reduction in the impulse generated by the anterior-posterior GRF in mixed-single steppers. Barrett et al. [12] reported that the main mechanism underlying the adaptation in balance recovery responses to repeated forward loss of balance in older adults using the same protocol as the present study was related improved control of centre of mass motion. A likely reason that BoS related parameters were also found to be mechanisms underlying adaptation in the present study, but not in the study by Barrett et al. [12], was that Barrett et al. [12] did not distinguish between subgroups, which would be expected to exhibit different adaptive behaviours in response to repeated forward loss of balance. The observed adaptations in BoS related parameters in the present study are also broadly consistent with those reported as a result of a 14-week stability-based training intervention in older adults designed to exercise the mechanisms for dynamic stability [17]. Taken together these findings suggest the ability of older adults to adapt from a multiple to single step recovery strategy following repeated exposure to forward loss of balance is due to a combination of taking longer, more rapid steps, as well as improved control of whole body centre of mass motion.

The main limitation of the present study was that adaptation in recovery responses may not have reached steady state within the number of trials performed. It therefore remains possible that further task exposure may have resulted in more participants adapting from a multiple to a single step strategy and those who did may have experienced greater improvements in MoS. Further, a degree of caution is also warranted in generalising the findings of the present study due to the task specificity of different types of falls [18]. Finally, the assumption underlying the concept of MoS that the excursion of the centre of mass is small relative to the length of the pendulum is violated during balance recovery by stepping [12]. However this does not alter our conclusion that adaptive stepping responses that favoured improved MoS were evident in our study.

### Conclusion

Older adults that exhibited a change from a multiple to a single step recovery strategy following repeated exposure to forward loss of balance were found to have improved dynamic stability at FC in the trials where they were able to recover with single compared to multiple steps. Improvements in dynamic stability were due to taking longer and more rapid steps and a reduced forward velocity of the whole body centre of mass, and were consistent with differences in stepping behaviour between exclusively single and exclusively multiple steppers. The observed adaptations occurred within a single test session and suggest older adults that are close to the threshold of successful recovery can rapidly improve dynamic stability following repeated exposure to a forward loss of balance.
